# Role of rs2366152 single-nucleotide variant located in the long noncoding RNA HOTAIR gene in the cervical cancer susceptibility in a Polish population

**DOI:** 10.1007/s13353-023-00822-3

**Published:** 2023-12-29

**Authors:** Sebastian Łaźniak, Anna Sowińska, Andrzej Roszak, Margarita Lianeri, Andrzej Pławski, Adrianna Mostowska, Paweł Piotr Jagodziński

**Affiliations:** 1 Department of Biochemistry and Molecular Biology, Poznań University of Medical Sciences, 6 Święcickiego St., 60-781 Poznań, Poland; 2 Department of Computer Science and Statistics, Poznań University of Medical Sciences Poznań, Poznań, Poland; 3 Department of Radiotherapy and Gynecological Oncology, Greater Poland Cancer Center, Poznań, Poland; 4 Department of Electroradiology, Poznań University of Medical Sciences, Poznań, Poland; 5 Institute of Human Genetics, Polish Academy of Sciences, 60-479, Poznan, Poland

**Keywords:** Cervical cancer, Single nucleotide variant

## Abstract

Previous studies have demonstrated an association of the NC_000012.12:g.53962605A > G, (rs2366152) single-nucleotide variant (SNV) situated in the long noncoding homeobox transcript antisense intergenic RNA (HOTAIR) gene with HPV16-related cervical cancer pathogenesis. However, little is known about the role of rs2366152 in cervical cancer progression and how oral birth control pills use, parity, menopausal status, and cigarette smoking influence the role of rs2366152 in cervical carcinogenesis. HRM analysis was used to determine the rs2366152 SNV prevalence in patients with cervical squamous cell carcinoma (SCC) (*n* = 470) and control group (*n* = 499) in a Polish Caucasian population. Logistic regression analyses were adjusted for age, using birth control pills, parity, menopausal status, and cigarette smoking. Our genetic studies revealed that the G/A vs. A/A (*p* = 0.031, *p* = 0.002) and G/A + G/G vs. A/A (*p* = 0.035, *p* = 0.003) genotypes of rs2366152 SNV were significantly related to the grade of differentiation G3 and tumor stage III, respectively. Moreover, cervical cancer risk increased among patients with rs2366152 SNV who smoked cigarettes and used birth control pills. We conclude that rs2366152 may promote the invasion and rapid growth of cervical SCC. Moreover, rs2366152 with cigarette smoking and using birth control pills can also be a risk factor for cervical cancerogenesis.

## Introduction

Cervical cancer (CC) is the fourth cause of cancer-related death among women globally and the fourth most common cancer globally, with approximately 604,000 newly diagnosed cancer and 342,000 deaths in 2020 (Sung et al. 2021). In Poland, current estimates indicate 3862 cases of CC yearly and 2137 deaths. CC arrays 6th in prevalence among women in Poland and 3rd among women aged 15 to 44 (Poland, Human Papillomavirus and Related Cancers, Fact Sheet 2023).

CC is preceded by precursor lesions, classified histologically as cervical intraepithelial neoplasia (CIN) (Kornovski et al. 2021). CC develops slowly and does not cause any symptoms for many years; clear signs usually indicate an advanced stage that is difficult or almost impossible to cure (Fowler et al. 2023). The most common cervical cancer type is squamous cell carcinoma (SCC), which is responsible for about 80% of all cervical malignancies, while approximately 10% of cervical cancers are adenocarcinoma (Tanaka et al. 2021; Fowler et al. 2023). The remaining are rare types of cancer, such as adenosquamous carcinoma, carcinoid, and non-epithelial tumors (sarcomas) (Tanaka et al. 2021; Fowler et al. 2023). SCC is characterized by low histological differentiation, extensive local infiltration, rapid growth, and a tendency to metastasise to lymph nodes, lungs, bone, liver, and other organs (Butt and Botha 2019; Zhou and Peng 2020; Fowler et al. 2023).

Human papillomavirus (HPV), particularly types 16 and 18, is believed to be the leading etiological agent of cervical cancerogenesis (Gutierrez-Xicotencatl et al. 2021). The HPV oncogenic potential is determined mainly by two virus genes encoding proteins E6 and E7 (Tomaić 2016). These proteins in viruses with high oncogenic potential are called oncoproteins. E6 and E7 oncoproteins interact with host cell cycle regulators, disrupting their function and leading to genetic instability, malignant transformation, and tumor development (Tomaić 2016). Carcinogenesis in the cervix is a multi-complex process in which, apart from oncogenic viruses, hereditary and environmental factors are also involved (Maciag and Villa 1999; Bowden et al. 2021). CC is a cancer whose development has a significant genetic background (Bowden et al. 2021). The disease risk is much higher in women with a first-degree relative with CC (Zoodsma et al. 2004). The genetic factors influencing CC development can be potentiated by coinfection with the human immunodeficiency virus or type 2 herpes simplex virus, high parity, pollutants, cigarette smoking, long-term, birth control pills use, and menopause status (Quinlan 2021; Tekalegn, et al. 2022).

The largest genome-wide association study (GWAS) meta-analysis recently revealed that the *GSDMB*, *HLA-DRB1*, *LINC00339*, *CLPTM1L*, *CDC42*, and *PAX8/PAX8-AS1* genes related to the immune response, growth of reproductive tract, and apoptosis/proliferation play a significant role in the development of CC (Koel et al. 2023). Various previously studied gene variants did not achieve the GWAS significance threshold. However, they may affect the progression and invasion of cervical malignancies (Bahrami et al. 2018).

Previous studies demonstrated an association of the NC_000012.12:g.53962605A > G, (rs2366152) single-nucleotide variant (SNV) with HPV16-related cervical cancer pathogenesis (Saha et al. 2016). The rs2366152 is located in the long noncoding homeobox transcript antisense intergenic RNA (HOTAIR) gene, which is involved in developing many cancers (Liu et al. 2020).

The influence of rs2366152 in cervical malignancies progression is still unclear. Little is known about how menopausal status, birth control pills use, parity, and cigarette smoking modify the influence of rs2366152 in the development of CC.

We determined the rs2366152 SNV distribution in women with cervical SCC in the Caucasian population from Poland. We also determined the frequency of rs2366152 in the differentiation grades of SCC and stages I–IV. The distribution of rs2366152 genotypes was also assessed in patients and controls subdivided into groups of menopausal status birth control pills use, parity, and cigarette smoking.

## Materials and methods

### Cases and control group

There were 470 patients with established cervical SCC and 499 healthy women controls enrolled in this study.

The patient’s grade of differentiation and stage was assessed based on the International Federation of Gynecology and Obstetrics (FIGO) classification system and the World Health Organization (WHO) (Saleh et al. 2020) (Table 1). Clinical characteristics of patients were gained from cases enrolled between May 2016 and January 2021 at the Department of Radiotherapy of the Greater Poland Cancer Center in Poznań, Poland. The controls, healthy women were arbitrarily obtained during medical examinations at the Heliodor Swiecicki Clinical Hospital, Poznan, Poland (Table 1). Data on active cigarette smoking within the last 12 months, birth control pills use, menopausal status, and parity were collected from cases and healthy women. Both cervical SCC patients and healthy controls were Caucasian individuals from Poland. All participants provided written and oral consent. The research methodology was approved by the Local Ethical Committee of the Poznań University of Medical Sciences (reference number of ethical approval: 1010/07).

**Table 1:** Clinical and demographic characteristics of patients with squamous cell carcinoma and controls

Characteristic	Patients (*n* = 470)	Controls (*n* = 499)	*p*
^a^Mean age (years) ± SD	52.5 ± 9.7	51.8 ± 9.5	0.484^c^
Tumor stage			
IA	62 (13.2%)		
IB	61 (13.0%)		
IIA	59 (12.6%)		
IIB	51(10.9%)		
IIIA	161 (34.3%)		
IIIB	52 (11.1%)		
IVA	11 (2.3%)		
IVB	13 (2.8%)		
Histological grade			
G1	91 (19.4%)		
G2	149 (31.7%)		
G3	101 (21.5%)		
Gx	129 (27.4%)		
Parity			
Never	52 (11.1%)	57 (11.4%)	0.859^b^
Ever	418 (88.9%)	442 (88.6%)	
Birth control pill use			
Never	253 (53.8%)	281 (56.3%)	0.437^b^
Ever	217 (46.2%)	218 (43.7%)	
Cigarette smoking			
Never	299 (63.6%)	329 (65.9%)	0.451^b^
Ever	171 (36.4%)	170 (34.1%)	
Menopausal status			
Premenopausal	165 (35.1%)	189 (37.9%)	0.371^b^
Postmenopausal	305 (64.9%)	310 (62.1%)	
HPV genotypes			
16 and 18 16, 18, 31, 33, 35, 39, 45, 51, 52, 56, 58, 59, and 68	321 (68.3%) 361 (76.8%)		

^a^Age at first diagnosis

^b^chi-squared

^c^Mann-Whitney test

## Methods

### Genotype determination

DNA was obtained from peripheral blood leucocytes via salting out cellular proteins. The primers were designated employing Beacon Designer 7.9 PREMIER Biosoft International San Francisco, CA. The 160 bp DNA fragment including the A > G rs2366152 SNV was amplified using the primers (forward 5′ TATATTCTGTGAGTTGTGTT 3′ and reverse 5′ ATCAGCAGATGGAGATTA′). The genotype of rs2366152 was determined via high-resolution melting (HRM) curve analysis using 5 × HOT FIREPol EvaGreen Mix no ROX (Solis BioDyne, Tartu, Estonia) with a LightCycler 480 system (Roche Diagnostics, Mannheim, Germany). Randomly, 10% of this variant’s patients and healthy control samples were checked by Sanger sequencing analyses. The agreement rate between HRM and Sanger sequencing results was 100%.

### Statistical assessment

The genotype frequency in the women with cervical SCC and healthy controls and their genotype departure from Hardy-Weinberg (HW) equilibrium were assessed using a chi-squared test. The rs2366152 SNV was evaluated for risk factors of cervical SCC using the Cochran-Armitage *p*-trend test (*p*_trend_). The chi-squared and Fisher exact tests were employed to find the divergencies in genotypic frequencies between the women with cervical SCC and healthy controls. The odds ratio (OR) and 95% confidence intervals (95% CI) were also determined. A logistic regression analysis was employed to adjust for menopausal status, using birth control pills, parity, tobacco smoking, and age. A *p* value greater than 0.05 means that no effect was observed.

## Results

### Prevalence of the rs2366152 SNV among all cases and healthy controls

The chi-squared test values of HW equilibrium were 0.052 and 0.057 for the patients and healthy controls, respectively. The assessment of the rs2366152 genotype presence in patients and controls is stated in Table 2. We did not find the statistical value *p*_trend_ = 0.850 calculated for the rs2366152 with all patients. However, the logistic regression analysis adjusted for the menopausal status, birth control pills use, parity, cigarette smoking, and age showed an influence of rs2366152 on the development of cervical SCC (Table 2). For G/A vs. A/A, the adjusted OR was 1.637 (95% CI 1.220–2.196, *p* = 0.001); for G/A + G/G vs. A/A, the adjusted OR was 1.522 (95% CI 1.153–2.010, *p* = 0.003). However, for G/G vs. A/A, we did not show a contribution of rs2366152; the adjusted OR was 1.168 (95% CI 0.704–1.935, *p* = 0.548).

**Table 2:** Prevalence of the *HOTAIR A* > *G (*rs2366152*)* polymorphism among patients with squamous cell carcinoma and controls

Genotype	Patients (frequency %)	Controls (frequency %)	Odds ratio (95%CI)	*p*^a^	Adjusted odds ratio (95%CI)^c^	*p*	*p*_trend_
ALL							
A/A	194 (41.3)	225 (45.1)	Referent		Referent		0.850
G/G	42 (8.9)	37 (7.4)	1.317 (0.813–2.132)	0.262	1.168 (0.704–1.935)	0.548	
G/A	234 (49.8)	237 (47.5)	1.145 (0.880–1.491)	0.314	**1.637 (1.220–2.196)**	**0.001**	
G/A + G/G	276 (58.7)	274 (54.9)	1.168 (0.905–1.507)	0.231	**1.522 (1.153–2.010)**	**0.003**	
MAF^d^	0.34	0.31					
Tumor stage							
IA + IB							
A/A	56 (45.5)	225 (45.1)	**Referent**	−	Referent	−	0.969
G/G	9 (7.3)	37 (7.4)	0.977 (0.446–2.143)	1.000^b^	1.530 (0.537–4.362)	0.426	
G/A	58 (47.2)	237 (47.5)	0.983 (0.653–1.482)	0.936	0.938 (0.555–1.689)	0.908	
G/A + G/G	67 (54.5)	274 (54.9)	0.983 (0.661–1.460)	0.931	1.305 (0.785–2.171)	0.304	
MAF^d^	0.31	0.31					
IIA + IIB							
A/A	52 (47.3)	225 (45.1)	Referent	−	Referent	−	0.990
G/G	7 (6.3)	37 (7.4)	0.819 (0.346–1.939)	0.834^b^	1.751 (0.513–5.975)	0.371	
G/A	51 (46.4)	237 (47.5)	0.931 (0.607–1.427)	0.743	0.987 (0.604–1.612)	0.957	
G/A + G/G	58 (52.7)	274 (54.9)	0.916 (0.606–1.385)	0.677	1.145 (0.718–1.827)	0.569	
MAF^d^	0.30	0.31					
IIIA + IIIB							
A/A	75 (35.5)	225 (45.1)	Referent	−	Referent	−	0.625
G/G	23 (10.9)	37 (7.4)	**1.865 (1.042–3.339)**	**0.034**	2.012 (0.943–4.291)	0.071	
G/A	113 (53.6)	237 (47.5)	**1.430 (1.014–2.018)**	**0.041**	**5.248 (2.742–10.05)**	**0.002**	
G/A + G/G	136 (64.5)	274 (54.9)	**1.489 (1.068–2.077)**	**0.019**	**4.311 (2.473–7.516)**	**0.003**	
MAF^d^	0.38	0.31					
IVA + IVB							
A/A	11 (42.3)	225 (45.1)	Referent	−	Referent	−	0.664
G/G	3 (11.5)	37 (7.4)	1.658 (0.442–6.230)	0.436^b^	2.482 (0.518–11.88)	0.255	
G/A	12 (46.2)	237 (47.5)	1.036 (0.448–2.395)	0.935	2.216 (1.547–3.085)	0.134	
G/A + G/G	15 (57.7)	274 (54.9)	1.120 (0.504–2.487)	0.781	11.32 (3.483–36.81)	0.061	
MAF^d^	0.35	0.31					
Differentiation grade							
G1							
A/A	34 (37.4)	225 (45.1)	Referent	−	Referent	−	0.235
G/G	9 (9.9)	37 (7.4)	1.610 (0.714–3.629)	0.253^b^	1.237 (0.633–2,.416)	0.533	
G/A	48 (52.7)	237 (47.5)	1.340 (0.833–2.157)	0.227	2.021 (1.531–2.023)	0.627	
G/A + G/G	57 (62.6)	274 (54.9)	1.377 (0.869–2.181)	0.172	0.996 (0.519–1.910)	0.990	
MAF^d^	0.36	0.48					
G2							
A/A	68 (45.6)	225 (45.1)	Referent	−	Referent	−	0.703
G/G	9 (6.0)	37 (7.4)	0.805 (0.370–1.751)	0.706^b^	1.155 (0.425–3.134)	0.778	
G/A	72 (48.3)	237 (47.5)	1.005 (0.689–1.468)	0.979	0.975 (0.638–1.488)	0.906	
G/A + G/G	81 (54.4)	274 (54.9)	0.978 (0.677–1.413)	0.906	1.158 (0.773–1.735)	0.478	
MAF^d^	0.30	0.48					
G3							
A/A	44 (43.6)	225 (45.1)	Referent	−	Referent	−	0.940
G/G	8 (7.9)	37 (7.4)	1.106 (0.482–2.535)	0.829^b^	1.027 (0.395–2.667)	0.357	
G/A	49 (48.5)	237 (47.5)	1.057 (0.677–1.652)	0.807	**2.126 (1.601–4.264)**	**0.031**	
G/A + G/G	57 (56.4)	274 (54.9)	1.064 (0.691–1.637)	0.777	**1.929 (1.048–3.549)**	**0.035**	
MAF^d^	0.32	0.48					
Gx							
A/A	48 (37.2)	225 (45.1)	Referent	−	Referent	−	0.254
G/G	16 (12.4)	37 (7.4)	**2.027 (1.043–3.939)**	**0.035**	3.703 (1.378–9.952)	0.091	
G/A	65 (50.4)	237 (47.5)	1.286 (0.849–1.948)	0.235	2.088 (1.142–2.340)	0.341	
G/A + G/G	81 (62.8)	274 (54.9)	1.389 (0.931–2.064)	0.108	4.421 (6.338–7.543)	0.171	
MAF^d^	0.38	0.48					

^a^*χ*^2^ or ^b^Fisher exact test

^c^ORs were adjusted by age, menopausal status, birth control pill use, parity, and cigarette smoking

^d^Minor allele frequency

Significant results are highlighted in bold font. *NA*, the number of genotypes is too small; therefore, the logistic regression was not applied

### The rs2366152 genotype differences among patients with differentiation grades and different tumor stages

On stratifying cases according to differentiation grade and tumor stage, we showed the influence of rs2366152 in grade G3 and stage III (Table 2). In patients with stage III, the *p*-trend value assessed for the rs2366152 was not statistically significant (*p*_trend_ = 0.625). However, the logistic regression analysis, adjusting for the menopausal status, birth control pills use, parity, cigarette smoking, and age in patients with stage III, showed a contribution of rs2366152 to cervical malignancies (Table 2). For G/A vs. A/A, the adjusted OR was 5.248 (95% CI 2.742–10.05, *p* = 0.002); for G/A + G/G vs. A/A, the adjusted OR was 4.311 (95% CI 2.473–7.516, *p* = 0.003). However, we did not show a statistical contribution of rs2366152 for G/G vs. A/A, and the adjusted OR was 2.012 (95% CI 0.943–4.291, *p* = 0.071) (Table 2).

In patients with differentiation grade G3, the *p*-trend value assessed for rs2366152 SNV was not statistically significant (*p*_trend_ = 0.940). However, in the same patient group, after adjusting for confounders, we showed the G/A vs. A/A genotype as risk cervical SCC, with an adjusted OR = 2.126 (95% CI = 1.601–4.264, *p* = 0.031) as well as for G/A + G/G vs. A/A with an adjusted OR = 1.929 (95% CI = 1.048–3.549, *p* = 0.035) (Table 2). In this group of patients, we did not show the risk effect of the G/G vs. A/A genotype with an adjusted OR = 1.027 (95% CI = 0.395–2.667, *p* = 0.357).

The logistic regression analysis did not indicate the contribution of rs2366152 to tumor stages I, II, and IV and the grade of differentiation grades G1, G2, and GX (Table 2).

### The frequency of rs2366152 among women with cervical SCC and the control group with menopausal status, a history of birth control pills use, parity, and cigarette smoking

Stratification of patients for confounders showed the contribution of rs2366152 to patients with cigarette smoking and birth control pills (Table 3).

**Table 3:** The distribution of *HOTAIR A* > *G* (*rs2366152*) genotypes among squamous cell carcinoma risks: parity, oral contraceptive use, tobacco smoking, and menopausal status

High-risk exposure	Patients		Controls		Odds ratio (95% CI)	*p*	Adjusted odds ratio (95% CI)	*p*
Genotype	Ever	Never	Ever	Never	Ever		Never	
Parity								
A/A	173	21	199	26	Referent		Referent	
G/G	38	4	32	5	1.329 (0.793–2.226)	0.283	0.995 (0.234–4.221)	0.994
G/A	207	27	211	26	1.121 (0.847–1.483)	0.425	1.285 (0.580–2.845)	0.537
G/A + G/G	245	31	243	31	1.150 (0.878–1.507)	0.312	1.268 (0.589–2.726)	0.543
Birth control pill use								
A/A	82	112	97	128	Referent		Referent	
G/G	34	8	18	19	1.894 (0.976–3.675)	0.059	0.687 (0.312–1.520)	0.356
G/A	137	97	103	134	**1.627 (1.092–2.422)**	**0.017**	0.836 (0.579–1.207)	0.339
G/A + G/G	171	105	121	153	**1.652 (1.127–2.424)**	**0.010**	0.815 (0.570–1.163)	0.259
Smoking								
A/A	71	123	77	148	Referent		Referent	
G/G	12	30	12	25	**3.023 (1.448–6.311)**	**0.015**	0.322 (0.134–0.777)	0.0716
G/A	88	146	81	156	**2.178 (1.758–2.833)**	**0.043**	0.735 (0.536–1.009)	0.067
G/A + G/G	100	176	93	181	**10.490 (5.322–20.67)**	**0.001**	0.699 (0.512–0.955)	0.074
Menopausal status								
	Premenopausal	Postmenopausal	Premenopausal	Postmenopausal	Premenopausal		Postmenopausal	
A/A	57	137	85	140	Referent		Referent	
G/G	22	20	13	24	1.055 (0.422–2.641)	0.908	1.313 (0.732–2.355)	0.361
G/A	86	148	91	146	1.023 (0.625–1.675)	0.927	1.191 (0.862–1.643)	0.289
G/A + G/G	108	168	104	170	1.065 (0.664–1.712)	0.793	1.216 (0.891–1.661)	0.218

All *p* values were adjusted by age. Significant results are highlighted in bold font

In patients with cigarette smoking, we observed an influence of rs2366152 with cervical SCC development. The age standardization OR for women with cigarette smoking for G/G vs. A/A was 3.023 (95% CI = 1.448–6.311, *p* = 0.015), for G/A vs. A/A was 2.178 (95% CI = 1.758–2.833, *p* = 0.043), and for A/G + G/G vs. A/A, the age standardization OR was 10.490 (95% CI = 5.322–20.67, *p* = 0.001).

The age standardization OR for women with birth control pills use for G/A vs. A/A was 1.627 (95% CI = 1.092–2.422, *p* = 0.017), and for A/G + G/G vs. A/A, the age standardization OR was 1.652 (95% CI = 1.127–2.424, *p* = 0.010). In this group, we did not show rs2366152 as a risk of cervical SCC for G/G vs. A/A was 1.894 (95% CI = 0.976–3.675, *p* = 0.059). We also did not find rs2366152 as a risk of cervical SCC in the patient’s group divided according to menopausal status and parity.

## Discussion

HOTAIR is a non-protein-coding transcript longer than 200 nucleotides that belongs to an oncogenic-acting long noncoding RNA (lncRNA) (Tang and Hann 2018). HOTAIR is a trans-acting lncRNA, via interactions with various cellular partners, that governs gene expression processes on the epigenetic, transcriptional, or posttranscriptional level that regulate basic biochemical and cellular processes (Hajjari and Salavaty 2015; Tang and Hann 2018). HOTAIR is an oncogenic factor, and abnormal HOTAIR expressions are involved in the malignant transformation and development of at least 24 types of solid tumors (An and Liu 2022). HOTAIR lncRNA’s dysfunction promotes cancer cells’ proliferation, invasion, survival, metastasis, and drug resistance (He et al. 2022; Liang and Peng 2022; Wang et al. 2022). Deregulation of HOTAIR lncRNA expression can be a biomarker of poor prognosis and cancer progression for several solid tumors such as ovarian, gastric, and lung cancer (Shehata et al. 2020; Xin et al. 2021). Increased transcription of HOTAIR in various cancerous tissues is accompanied by shorter overall survival and disease-free survival, metastasis, and tumor resistance to chemo/radiotherapy (Tang et al. 2019; Lu et al. 2018; Raju et al. 2023). Recent studies revealed many HOTAIR sequence variations, mainly including SNVs in the HOTAIR gene (Liu et al. 2020). These genetic HOTAIR variants influence the expression and function of HOTAIR (Li et al. 2017). Most of the SNVs identified in the HOTAIR gene are responsible for acquiring oncotic HOTAIR activity, which supports the development of various solid tumors (Qi et al. 2016; Tian et al. 2016; Ge et al. 2017; Li et al. 2018; Moazeni-Roodi et al. 2020; Janaththani et al. 2021).

To date, it has been demonstrated that several HOTAIR gene SNPs are associated with the appearance and progression of lung, ovarian, breast, and gastric cancers, as well as cervical cancer (Qiu et al. 2017; Jin et al. 2017; Wang et al. 2019; Moazeni-Roodi et al. 2020; Ke et al. 2022; Liu et al. 2022, Yalınbaş Kaya et al. 2022).

The three SNVs in the HOTAIR gene, rs7958904, rs920778, and rs4759314, have been reported as risk factors appearance of cervical cancer in the Chinese population (Jin et al. 2017; Liu et al. 2022). Moreover, it has also been demonstrated that lncRNAs HOTAIR SNP rs920778 can predict cancer recurrence and worse overall survival (Weng et al. 2018). Additionally, Saha et al. (2016) demonstrated rs2366152G over-presentation in low HOTAIR-expressing HPV-positive cervical cancer cases compared to HPV-negative controls.

Our studies did not find an association of rs2366152 with the diagnosis of cervical SCC. Our observations agree with other genetic studies in the Chinese population, which also failed to present rs2366152 as a genetic risk factor for cervical SCC (Liu et al. 2022).

However, our genetic studies demonstrated the contribution of lncRNAs HOTAIR SNP rs2366152 to grade differentiation G3 and stages III of cervical malignancies. These findings may indicate that the G variant of s2366152 promotes the invasion of cervical malignancies cells to neighboring tissues and increase tumor growth compared to lower-grade cervical malignancies.

Evaluating confounding variables such as age, birth control pill use, parity, cigarette smoking, and menopause demonstrated that some were correlated with HOTAIR SNP rs2366152 in the diagnosis of cervical SCC. We observed a contribution of rs2366152 to cervical SCC in women with a positive history of using birth control pills and cigarette smoking. Our observation confirms previously demonstrated tobacco smoking and using birth control pills as environmental factors, which, alone or with genetic factors, may modulate the risk for the appearance of cervical SCC (Quinlan 2021; Tekalegn et al. 2022).

Our investigation is the first to present rs2366152 SNV as a new genetic risk factor for cervical malignant transformation in Polish women with a positive history of using birth control pills and cigarette smoking. We also find that the rs2366152 G variant supports the growth and invasion of malignant cells to surrounding tissue. To reinforce the value of our observations, these genetic studies should also be performed in other populations.
